# A Panel of Plasma Biomarkers for Differential Diagnosis of Parkinsonian Syndromes

**DOI:** 10.3389/fnins.2022.805953

**Published:** 2022-02-17

**Authors:** Qi Li, Zhen Li, Xiaoxuan Han, Xiao Shen, Fei Wang, Lipeng Bai, Zhuo Li, Rui Zhang, Yanlin Wang, Xiaodong Zhu

**Affiliations:** Department of Neurology, Tianjin Neurological Institute, Tianjin Medical University General Hospital, Tianjin, China

**Keywords:** Parkinsonian syndromes, Parkinson’s disease, biomarker, neurofilament light chain, panel

## Abstract

**Objective:**

The aim of our study is to explore the most reliable panel of plasma biomarkers for differential diagnosis of parkinsonian syndromes (PDSs). We selected five kinds of neurodegenerative proteins in plasma: neurofilament light chain (NfL), α-synuclein (α-syn), total tau, β-amyloid 42 (Aβ42) and β-amyloid 40 (Aβ40), and investigated the diagnostic value of these biomarkers.

**Methods:**

A total of 99 plasma samples from patients with Parkinson’s disease (PD), multiple system atrophy (MSA), progressive supranuclear palsy, and age-matched healthy controls (HCs) were enrolled in our study. Plasma NfL, α-syn, total tau, Aβ42, and Aβ40 levels were quantified by ultrasensitive single molecule array immunoassay. We used logistic regression analyses to examine diagnostic accuracy of these plasma biomarkers. Disease severity was assessed by the modified Hoehn and Yahr staging scale, Unified Parkinson’s Disease Rating Scale part III (UPDRS III), and the Mini-Mental State Examination (MMSE), and subsequently, correlation analysis was performed.

**Results:**

A combination of α-syn, Aβ42, Aβ40, Aβ42/40, and NfL could achieve a best diagnostic value in differentiating PDSs from HC and PD from HC, with an AUC of 0.983 and 0.977, respectively. By adding NfL to measurements of α-syn or Aβ42 or Aβ40 or Aβ42/40, the best discriminating panel was formed in differentiating atypical parkinsonian disorder (APD) and HC, and the discriminatory potential could reach a sensitivity of 100% and specificity of 100% (AUC = 1.000). For further distinguishing PD from APD, we found a combination of NfL, Aβ42, and total tau was the most reliable panel with equally high diagnostic accuracy. With respect to differentiating the subtypes of APD from one another, our results revealed that measurement of NfL, total tau, Aβ42, Aβ40, and Aβ42/40 was the best discriminating panel. Correlation analysis suggests that plasma Aβ42 levels were positively correlated to UPDRS part III scores in MSA. In terms of cognitive function, there was a relationship between plasma Aβ42/40 level and MMSE scores in patients with APD.

**Conclusion:**

In our study, various combinations of plasma biomarkers have great potentialities in identifying PDSs, with important clinical utility in improving diagnostic accuracy. Plasma NfL may have added value to a blood-based biomarker panel for differentiating PDSs.

## Introduction

Parkinson’s disease (PD) is one of the most common neurodegenerative disorders. Because of the large overlap of clinical symptoms, characterized by bradykinesia in combination with rest tremor, rigidity, or both (Geut et al., 2020), it is difficult to differentiate PD from atypical parkinsonian disorders (APDs) in early stages, which are mainly involved in multiple system atrophy (MSA) and progressive supranuclear palsy (PSP). There is an urgent need for reliable biomarkers to achieve an early and effective differential diagnosis in parkinsonian syndromes (PDSs).

Key factor that complicates the differentiation among different PDSs is the accompanying pathology. Generally, the neuropathologies of PDSs can be classified into two clusters, α-synucleinopathy and tauopathy (Geut et al., 2020). It is universally acknowledged that a definite diagnosis with neuropathological confirmation is difficult to achieve. Therefore, biofluid biomarkers are the topical issues for the differential diagnoses. Several proteins associated with neurodegeneration, such as neurofilament light chain (NfL), α-synuclein (α-syn), total tau, β-amyloid 42 (Aβ42) and β-amyloid 40 (Aβ40), have been wildly studied as potential biomarkers for PDSs in cerebrospinal fluid (CSF) (Marques et al., 2019; Parnetti et al., 2019; Oosterveld et al., 2020). It is valuable to investigate a convenient blood-based biomarker panel for differential diagnosis in PDSs.

The aim of this study was to investigate whether plasma NfL, α-syn, total tau, Aβ42, Aβ40, and Aβ42/Aβ40 contribute to an optimal biomarker panel to differentiate the subtypes of PDSs from one another and controls, quantified by an ultrasensitive single molecule array (Simoa) immunoassay. Furthermore, we performed the correlation analysis of plasma biomarkers with clinical features of disease severity in patients with PD and APD.

## Materials and Methods

### Study Participants

All participants were recruited from the movement disorders outpatient clinics of the General Hospital of Tianjin Medical University. We analyzed a total of 99 plasma samples from patients with PD (*n* = 45), MSA (*n* = 13), PSP (*n* = 8), and age-matched healthy controls (*n* = 33). Inclusion criteria for PD met the United Kingdom Parkinson’s Disease Society Brain Bank (UKPDSBB) criteria (Hughes et al., 1992). We defined patients entering the study as having MSA, following the “Second consensus statement on the diagnosis of MSA” (Gilman et al., 2008), and diagnosis of PSP patients was established according to the National Institute for Neurological Disorders and Stroke/Society criteria for PSP (Osaki et al., 2004). HCs who were recruited from the local community at the same period and matched for age and education have no history of serious physical, neurological, psychiatric disorders, and alcohol or substance abuse. The research protocols were approved and performed in accordance with the guidelines of the Ethics Committee of the General Hospital of Tianjin Medical University. Written informed consent for scientific research purposes was obtained from all subjects.

### Clinical Evaluation

The motor symptoms of patients were evaluated using the modified Hoehn and Yahr staging scale (H&Y stage) (Hoehn and Yahr, 1967) and Unified Parkinson’s Disease Rating Scale part III (UPDRS III) (Goetz et al., 2008) as measurements of clinical parkinsonian severity, which was performed during off medication, more than 12 h after the last dose of dopaminergic therapy. Global cognitive function was examined with the Mini-Mental State Examination (MMSE).

### Measurement of Plasma Biomarkers

Fasting blood samples were collected by peripheral venous blood into 10-mL EDTA tubes and centrifuged (2,500*g* for 15 min) within 1 h after collection, aliquoted, and stored in polypropylene tubes at −80°C until analysis. The plasma levels of NfL, α-syn, total tau, Aβ42, and Aβ40 were measured by researchers who were blinded to the diagnosis and separately assayed via Simoa NfL Advantage kits (Quanterix, Lexington, MA, United States), Neurology 3-Plex A advantage kit (lot 502473), and α-syn discovery kit (Lot 502566) on the basis of manufacturer’s introductions and standard procedures.

### Statistical Analyses

All statistical analyses were performed with SPSS 22.0 (IBM, Inc., Armonk, NY, United States) and GraphPad Prism 9 (La Jolla, CA, United States). Shapiro–Wilk test was used to examine the Gaussian distribution of our data (*p* > 0.05). Numerical variables were displayed as mean ± SD. Comparisons of plasma biomarkers among different diagnostic groups were assessed by one-way analysis of variance (ANOVA), Kruskal–Wallis, and Mann–Whitney *U* test. Multiple linear regression analyses adjusted for age, sex, and disease duration were conducted to examine the differences among diagnostic groups. The correlation among plasma proteins and clinical outcomes was accessed by Spearman rank correlation analysis. The diagnostic accuracy was examined with receiver operating characteristic (ROC) curve analyses. To determine the optimal differentiating biomarker panel, we performed a binary logistic regression analysis. *P* < 0.05 was considered statistically significant.

## Results

### Clinical Characteristics

A total of 99 participants consisting of 45 patients with PD, 13 patients with MSA, 8 patients with PSP, and 33 normal control subjects were enrolled in this study. Table 1 summarizes the characteristics and specific demographic data for all participants. The mean age of the four groups had no significant difference from each other (*p* = 0.05). In patients with APD, the motor severity (UPDRS part III scores and H–Y stage) was significantly greater than that in PD (*p* < 0.01).

**TABLE 1:** Clinical characteristics of study participants.

Characteristics	PD (*n* = 45)	MSA (*n* = 13)	PSP (*n* = 8)	HC (*n* = 33)	*p-*value
Male, %	48.89	78.57	62.50	51.52	<0.01**
Age, y	66.15 ± 4.75	63.11 ± 7.69	70.00 ± 7.31	66.15 ± 4.75	0.05
Disease duration, y	4.92 ± 2.47	2.22 ± 0.67	2.33 ± 1.86	NA	<0.01**
Hoehn and Yahr stage	1.31 ± 0.53	3.63 ± 0.92	3.17 ± 0.41	NA	<0.01**
MDS-UPDRS III (off)	17.95 ± 10.70	42.54 ± 18.00	25.75 ± 9.87	NA	<0.01**
MMSE	28.43 ± 1.86	26.69 ± 2.10	25.00 ± 5.34	NA	<0.01**
NfL (pg/mL)	20.43 ± 14.09	86.53 ± 33.74	53.90 ± 22.97	16.00 ± 5.18	<0.01**
Total tau (pg/mL)	1.08 ± 0.66	0.51 ± 0.18	0.55 ± 0.17	0.93 ± 0.63	<0.01**
Aβ42 (pg/mL)	10.10 ± 2.99	5.30 ± 1.17	5.83 ± 2.28	5.26 ± 2.01	<0.01**
Aβ40 (pg/mL)	167.35 ± 49.41	119.50 ± 23.78	136.39 ± 30.76	103.90 ± 31.00	<0.01**
Aβ42/40	0.04 ± 0.01	0.04 ± 0.01	0.04 ± 0.01	0.05 ± 0.01	<0.01**
α-synuclein (pg/mL)	43.50 ± 33.63	44.01 ± 18.51	53.75 ± 52.18	10.36 ± 5.51	<0.01**

*Data are presented as mean ± SD. The p-values were obtained from comparisons among four groups of participants using analysis of variance.*

**p < 0.01.

*PD, Parkinson’s disease; MSA, multiple system atrophy; PSP, progressive supranuclear palsy; HC, healthy controls; MMSE, Mini-Mental State Examination; NfL, neurofilament light chain; Aβ42, β-amyloid 42; Aβ40, β-amyloid 40; UPDRS, Unified Parkinson’s Disease Rating Scale; NA, not available.*

### Plasma Biomarker Levels in Different Diagnostic Groups

**FIGURE 1:**
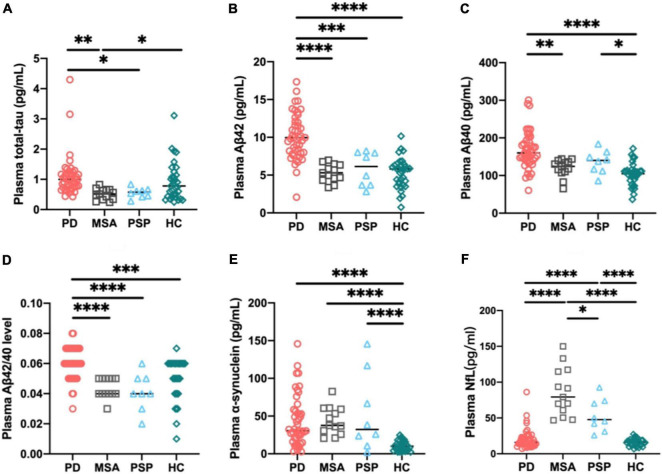
Individual plasma biomarker levels of normal controls and in different diagnostic groups. The plasma total tau **(A)**, Aβ42 **(B)**, and Aβ42/40 **(D)** levels were significantly increased in patients with PD compared with those in APD. The plasma Aβ40 **(C)**, Aβ42/40 **(D)**, and α-synuclein **(E)** levels were significantly higher in PD group compared with healthy controls. The plasma NfL **(F)** level was significantly increased in patients with MSA and PSP compared with PD and controls. *p*-values are from multiple linear regression models adjusting for age, sex, and disease duration. PD, Parkinson’s disease; MSA, multiple system atrophy; PSP, progressive supranuclear palsy; HC, healthy controls; NfL, neurofilament light chain; Aβ42, β-amyloid 42; Aβ40, β-amyloid 40; Aβ42/40, β-amyloid 42/40; ^∗∗∗∗^*p* < 0.0001, ^∗∗∗^*p* < 0.001, ^∗∗^*p* < 0.01, ^∗^*p* < 0.05.

Mean plasma biomarker levels among different diagnostic groups are presented in Table 1. We compared individual plasma biomarker levels of normal controls and in different diagnostic groups. To adjust for the potential confounding factors of age, sex, and disease duration, we performed multiple linear regression. After adjustment, we found that the plasma Aβ40, Aβ42/40, and α-syn levels were significantly higher in the PD group compared with HCs (Aβ40: PD vs. HC: *p* < 0.0001; Aβ42/40: PD vs. HC: *p* < 0.001; α-syn: PD vs. HC: *p* < 0.0001; Figures 1C–E). The levels of plasma total tau, Aβ42, and Aβ42/40 were significantly elevated in patients with PD compared with those in APD (total tau: PD vs. MSA: *p* < 0.01, PD vs. PSP: *p* < 0.05; Aβ42: PD vs. MSA: *p* < 0.0001, PD vs. PSP: *p* < 0.001; Aβ42/40: PD vs. MSA: *p* < 0.0001, PD vs. PSP: *p* < 0.0001; Figures 1A,B,D). Moreover, the level of plasma NfL was significantly increased in patients with MSA and PSP when compared with PD (PSP, *p* < 0.0001; MSA, *p* < 0.0001) and controls (PSP, *p* < 0.0001; MSA, *p* < 0.0001). Plasma NfL concentrations in patients with MSA were clearly higher than those in PSP (*p* = 0.02) (Figure 1F). Only patients with PSP had an increased expression level of Aβ40 and α-syn compared with controls (Aβ40: *p* < 0.05; α-syn: *p* < 0.0001, Figures 1C,E).

### Diagnostic Accuracy of Plasma Biomarkers

Discriminatory value of plasma NfL, total tau, Aβ42, Aβ40, Aβ42/40 and α-syn as a single biomarker and as part of plasma biomarker panel in differentiating PDSs (PD and APD) from HC is presented in Table 2. The biomarkers were selected to combine as a panel according to the level of AUC; that is, we selected the biomarkers to combine as a panel according to the ability of independently predicted diagnosis of individual biomarker.

**TABLE 2:** Discriminatory value of plasma NfL, total tau, Aβ42, Aβ40, Aβ42/40 and α-syn as a single biomarker and as part of plasma biomarker panel in differentiating PDS (PD and APD) from HC.

Diagnostic groups	Predictor	AUC	Sensitivity	Specificity
**PDS vs. HC**	**Single biomarker**			
	α-Syn	0.889	80.0%	93.9%
	Aβ40	0.824	83.1%	78.8%
	Aβ42	0.800	70.8%	87.9%
	NfL	0.694	46.2%	97.0%
	Aβ42/40	0.576	48.5%	51.5%
	Total tau	0.513	83.1%	33.3%
	**Biomarker panel**			
	Aβ42, Aβ40, Aβ42/40, NfL, and α-syn	0.983	98.5%	93.9%
	α-Syn, Aβ40, Aβ42/40, and NfL	0.982	96.9%	93.9%
	α-Syn, Aβ40, and NfL	0.980	93.8%	97.0%
	α-Syn, total tau, and NfL	0.921	73.8%	100%
**PD vs. HC**	**Single biomarker**			
	Aβ42	0.925	93.1%	87.9%
	α-Syn	0.875	73.3%	97.0%
	Aβ40	0.873	88.9%	78.8%
	Aβ42/40	0.736	53.3%	90.9%
	Total tau	0.612	42.4%	86.7%
	NfL	0.558	33.3%	87.9%
	**Biomarker panel**			
	Aβ42, Aβ40, Aβ42/40, NfL, and α-syn	0.977	97.8%	93.9%
	Aβ42, Aβ40, Aβ42/40, and α-syn	0.974	95.6%	97.0%
	Aβ42, Aβ40, and α-syn	0.972	95.6%	97.0%
**APD vs. HC**	**Single biomarker**			
	NfL	0.998	100%	97.0%
	α-Syn	0.920	90.3%	93.9%
	Aβ42/40	0.721	80%	81.8%
	Aβ40	0.715	70.6%	78.8%
	Total tau	0.711	75.0%	75.8%
	Aβ42	0.521	35.0%	76.8%
	**Biomarker panel**			
	NfL and α-syn	1.000	100%	100%
	NfL and Aβ40	1.000	100%	100%
	NfL and Aβ42	1.000	100%	100%
	NfL and Aβ42/40	1.000	100%	100%
	NfL and total tau	0.998	100%	97.0%
**MSA vs. HC**	**Single biomarker**			
	NfL	1.000	100%	100%
	α-Syn	0.990	100%	93.9%
	Total tau	0.737	66.7%	75.8%
	Aβ42/40	0.720	66.7%	75.8%
	Aβ40	0.667	83.3%	54.5%
	Aβ42	0.513	58.3%	57.6%
**PSP vs. HC**	**Single biomarker**			
	NfL	0.996	100%	100%
	α-Syn	0.814	100%	93.9%
	Aβ40	0.788	83.3%	54.5%
	Aβ42/40	0.723	66.7%	75.8%
	Total tau	0.670	66.7%	75.8%
	Aβ42	0.572	58.3%	42.4%
	**Biomarker panel**			
	NfL and α-syn	1.000	100%	100%
	NfL and Aβ40	1.000	100%	100%
	NfL and Aβ42	1.000	100%	100%
	NfL and Aβ42/40	1.000	100%	100%
	NfL and total tau	0.996	100%	97.0%

*PDS, parkinsonian syndrome; APD, atypical parkinsonian disorders; PD, Parkinson’s disease; MSA, multiple system atrophy; PSP, progressive supranuclear palsy; HC, healthy controls; NfL, neurofilament light chain; Aβ42, β-amyloid 42; Aβ40, β-amyloid 40; Aβ42/40, β-amyloid 42/40; AUC, area under the curve; α-syn, α-synuclein.*

#### Parkinsonian Syndromes vs. Healthy Controls

Plasma α-syn showed 80% sensitivity and 93.9% specificity with an AUC of 0.889 for differentiating PDSs (PD, MSA, and PSP) from HC, with the highest diagnostic value among individual biomarkers, whereas a combination of Aβ42, Aβ40, Aβ42/40, NfL, and α-syn could achieve a better diagnostic value in differentiating PDSs from HC with an AUC of 0.983 with 98.5% sensitivity and 93.9% specificity. However, the panel of α-syn, Aβ40, and NfL could achieve a relatively desired effect with the minimum types of markers (an AUC of 0.980 with a sensitivity and specificity of 93.8 and 97.0%). In order to differentiate PD subjects from HC, we found Aβ42 as the dominant single biomarker revealed an AUC of 0.925, with a sensitivity and specificity of 93.1 and 87.9%. There was very good discriminatory power between PD groups and HC with an AUC of 0.977 with a sensitivity and specificity of 97.8 and 93.9% using the panel of Aβ42, Aβ40, Aβ42/40, NfL, and α-syn, the same as the panel for discriminating PDSs and HC. The combination of Aβ42, Aβ40, and α-syn was also a good choice to discriminating PD from HC with the highest specificity up to 97% (AUC = 0.972). When looking into the APD (MSA and PSP) groups, NfL exhibited very good discriminatory power in differentiating MSA and PSP from HC with an AUC of 1.000 and 0.996, respectively. By adding NfL to measurement of α-syn or Aβ42 or Aβ40 or Aβ42/40, the best discriminating panel was formed in differentiating APD and HC with a sensitivity of 100% and specificity of 100% (AUC = 1.000; Table 2), implying NfL was the extraordinarily desirable biomarker for differentiating APD from HC.

#### Parkinson’s Disease vs. Atypical Parkinsonian Disorder

The ROC curve analysis revealed that NfL was the most reliable biomarker to differentiate PD from APD, which had a sensitivity of 88.9% and a specificity of 95.0% with an AUC of 0.963. When we looked into differentiating PD from MSA and PSP, NfL still exhibited very good discriminatory power with an AUC of 0.983 and 0.993, respectively (PD vs. MSA: a sensitivity and specificity of 100 and 95.6%, respectively; PD vs. PSP: a sensitivity and specificity of 82.5 and 100%, respectively). As seen from Table 3, the best differentiating panel between PD and APD was formed by the combination of plasma NfL, Aβ42, and total tau, which yielded an ideal sensitivity and specificity of 100% (AUC = 1.000), whereas the combination of Aβ42 and total tau without NfL revealed an AUC of 0.963 with a sensitivity of 95% and a specificity of 91.1%.

**TABLE 3:** Discriminatory value of plasma NfL, total tau, Aβ42, Aβ40, Aβ42/40 and α-syn as a single biomarker and as part of plasma biomarker panel in differentiating PD from APD (MSA and PSP).

Diagnostic groups	Predictor	AUC	Sensitivity	Specificity
**PD vs. APD**	**Single biomarker**			
	NfL	0.963	88.9%	95.0%
	Aβ42	0.924	71.1%	100%
	Total tau	0.914	80.0%	95.0%
	Aβ42/40	0.897	73.0%	100%
	Aβ40	0.791	73.3%	85.0%
	α-Syn	0.562	55.6%	65.6%
	**Biomarker panel**			
	NfL, Aβ42 and total tau	1.000	100%	100%
	NfL and total tau	0.996	95.6%	100%
	NfL, Aβ42 and Aβ42/40	0.990	100%	95.6%
	Aβ42, total tau, and Aβ42/40	0.981	100%	88.9%
**PD vs. MSA**	**Single biomarker**			
	NfL	0.983	100%	95.6%
	Aβ42	0.961	88.9%	100%
	Total tau	0.930	80.0%	100%
	Aβ42/40	0.896	77.8%	100%
	Aβ40	0.850	78.9%	100%
	α-Syn	0.600	55.6%	65.0%
	**Biomarker panel**			
	NfL, total tau, and Aβ42/40	1.000	100%	100%
	NfL and total tau	0.998	100%	97.8%
	Aβ42 and total tau	0.985	91.1%	100%
**PD vs. PSP**	**Single biomarker**			
	NfL	0.993	82.5%	100%
	Aβ42/40	0.897	73.3%	100%
	Total tau	0.892	82.2%	87.5%
	Aβ42	0.869	71.1%	100%
	Aβ40	0.703	62.2%	75.0%
	α-Syn	0.506	62.3%	37.5%
	**Biomarker panel**			
	NfL, Aβ42/40, and total tau	1.000	100%	100%
	NfL, total tau, and Aβ42	1.000	100%	100%
	NfL and total tau	0.992	95.6%	100%
	NfL and Aβ42/40	0.978	95.6%	100%
	NfL and Aβ42	0.967	93.3%	100%

*APD, atypical parkinsonian disorders; PD, Parkinson’s disease; MSA, multiple system atrophy; PSP, progressive supranuclear palsy; HC, healthy controls; NfL, neurofilament light chain; Aβ42, β-amyloid 42; Aβ40, β-amyloid 40; Aβ42/40, β-amyloid 42/40; AUC, area under the curve; α-syn, α-synuclein.*

#### Multiple System Atrophy vs. Progressive Supranuclear Palsy

Neurofilament light was the most reliable biomarker to differentiate MSA from PSP, which had a sensitivity of 78.3% and a specificity of 87.5% (AUC = 0.802; Table 4). The model of total tau, Aβ42, Aβ40, and Aβ42/40 could differentiate MSA from PSP with an AUC of 0.823. Now adding plasma NfL into the model, the best discriminatory panel was formed by the combination of NfL, total tau, Aβ42, Aβ40, and Aβ42/40 with an AUC of 1.000, a sensitivity of 100%, and a specificity of 100%.

**TABLE 4:** Discriminatory value of plasma NfL, total tau, Aβ42, Aβ40, Aβ42/40 and α-syn as a single biomarker and as part of plasma biomarker panel in differentiating MSA from PSP.

Diagnostic groups	Predictor	AUC	Sensitivity	Specificity
**MSA vs. PSP**	**Single biomarker**			
	NfL	0.802	78.3%	87.5%
	Aβ40	0.698	83.3%	62.5%
	Aβ42	0.594	100%	50.0%
	Total tau	0.583	82.2%	57.5%
	Aβ42/40	0.573	58.5%	63.8%
	α-Syn	0.551	50.0%	67.6%
	**Biomarker panel**			
	NfL, total tau, Aβ42, Aβ40, and Aβ42/40	1.000	100%	100%
	NfL, total tau, Aβ42, Aβ40, Aβ42/40, and α-syn	1.000	100%	100%
	NfL, total tau, Aβ42, and Aβ40	0.844	83.3%	87.5%
	Total tau, Aβ42, Aβ40, and Aβ42/40	0.823	91.7%	62.5%

*MSA, multiple system atrophy; PSP, progressive supranuclear palsy; HC, healthy controls; NfL, neurofilament light chain; Aβ42, β-amyloid 42; Aβ40, β-amyloid 40; Aβ42/40, β-amyloid 42/40; AUC, area under the curve; α-syn, α-synuclein.*

A summary of the best single biomarker and panel in comparing different PDSs from one another and controls is presented in Table 5.

**TABLE 5:** The best single biomarker and panel in comparing different parkinsonian syndromes with each other and controls.

Diagnostic groups	Single biomarker	AUC	Panel	AUC
PDS-HC	α-Syn	0.889	Aβ42, Aβ40, Aβ42/40, NfL, and α-syn	0.983
PD-HC	Aβ42	0.925	Aβ42, Aβ40, Aβ42/40, NfL, and α-syn	0.977
APD-HC	NfL	0.998	NfL and α-syn or Aβ42 or Aβ40 or Aβ42/40	1
MSA-HC	NfL	1	NA	NA
PSP-HC	NfL	0.996	NfL and α-syn or Aβ42 or Aβ40 or Aβ4240	1
PD-APD	NfL	0.963	NfL, Aβ42 and total tau	1
PD-MSA	NfL	0.983	NfL, Aβ42/40, and total tau	1
PD-PSP	NfL	0.993	NfL, total tau, and Aβ42/40 or Aβ42	1
MSA-PSP	NfL	0.802	NfL, total tau, Aβ42, Aβ40, and Aβ42/40	1

*PDS, parkinsonian syndrome; APD, atypical parkinsonian disorders; PD, Parkinson’s disease; MSA, multiple system atrophy; PSP, progressive supranuclear palsy; HC, healthy controls; NfL, neurofilament light chain; Aβ42, β-amyloid 42; Aβ40, β-amyloid 40; Aβ42/40, β-amyloid 42/40; AUC, area under the curve; α-syn, α-synuclein.; NA, not available.*

### Correlation Between Plasma Biomarkers and Motor Severity

**FIGURE 2:**
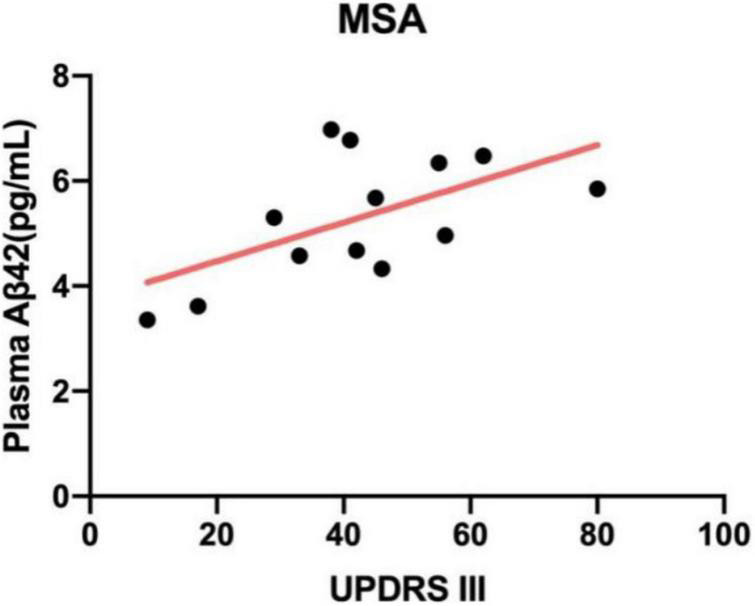
Correlation between plasma Aβ42 and UPDRS part III scores in patients with MSA. The levels of plasma Aβ42 were positively related to UPDRS part III (*r* = 0.589, *p* = 0.03). MSA, multiple system atrophy; Aβ42, β-amyloid 42; UPDRS part III, Unified Parkinson’s Disease Rating Scale part III.

We investigated whether there was a relationship between plasma biomarkers and motor severity as measured by MDS-UPDRS part III and H&Y stage. According to Table 1, patients with APD had significantly worse motor dysfunction compared with PD patients (*p* < 0.01 by ANOVA). Correlation analysis revealed that plasma Aβ42 levels were positively correlated to UPDRS part III scores in patients with MSA (*r* = 0.589, *p* = 0.03; Figure 2). Other biomarkers were not correlated with UPDRS part III during “off” time in PDS patients.

### Correlation Between Plasma Biomarkers and Cognitive Function

**FIGURE 3:**
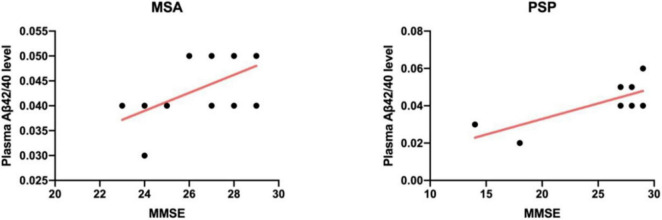
Scatter plot showing the relationship between plasma Aβ42/40 level and MMSE scores in the MSA group **(Left)** and PSP group **(Right)**. Solid line indicates a correlation observed between Aβ42/40 level and global cognitive status in patients with APD (MSA: *r* = 0.583, *p* = 0.03; PSP: *r* = 0.763, *p* = 0.03). MSA, multiple system atrophy; PSP, progressive supranuclear palsy; Aβ42/40, β-amyloid 42/40; MMSE, Mini-Mental State Examination.

Previous studies have confirmed that β-amyloid contributed to the prediction of cognitive deterioration in other neurodegenerative diseases, just as PD, not limited to Alzheimer disease (AD). We next investigated the relevance between plasma biomarkers and cognitive status (MMSE) in each diagnostic group. By means of correlation analysis, we found that plasma Aβ42/40 level positively correlated with MMSE scores only in patients with APD (MSA: *r* = 0.583, *p* = 0.03; PSP: *r* = 0.763, *p* = 0.03; Figure 3).

## Discussion

In this study, we aimed to demonstrate whether plasma levels of NfL, α-syn, total tau, Aβ42, Aβ40, and Aβ42/40 integrated could be applied to identify PDSs. For this purpose, we measured the levels of several specific and meaningful biomarkers in plasma quantified by high-sensitivity immunoassays. Our results indicated that plasma NfL was significantly increased, and plasma total tau, Aβ42, and Aβ42/40 levels were lower in patients with APD than those in PD. NfL is an extraordinarily prospective biomarker as a single measure for differentiating APD (MSA and PSP) from PD and HCs as well as differentiating MSA from PSP, with high sensitivity and specificity. By means of adding NfL to the biomarker panel, the diagnostic accuracy of dramatically improved on visual inspection. According to our results, various combinations of these six biomarkers were able to differentiate PDSs from HC, APD from PD, and the subtypes of APD from one another. Furthermore, we performed the correlation analysis suggesting that there was a correlation observed between plasma Aβ with motor and cognitive function in APD. Together, a measurement of integrated biomarker panel has great potentialities of differential diagnosis in PDSs, and these potential biomarkers may be indicators of monitoring motor and cognitive progression in patients with PDSs.

NfL is the most important and abundant subunit in neurofilaments. Under normal conditions, NfL is released from axons, probably in an age-dependent manner, and increases with age (Disanto et al., 2017). However, on special occasions in response to CNS axonal damage, such as inflammatory, neurodegenerative, traumatic, or vascular injury, the release of NfL is notably rising (Gaetani et al., 2019). Our findings are consistent with those reported in previous studies (Gaetani et al., 2019; Marques et al., 2019; Archer et al., 2020). All the relevant studies mentioned previously verified that plasma NfL was significantly higher in APD than that in PD and thus proved to have diagnostic value in identifying APD.

Definitely, plasma and CSF amyloid β (ratio of amyloid β42 to amyloid β40) reflect brain amyloidosis (Nakamura et al., 2018; Schindler et al., 2019). Aβ peptides are important for neuronal information processing and are major constituents of amyloid plaques deposited in the brains of patients with Alzheimer’s disease (AD), but also seen in other neurodegenerative diseases and notably in patients accompanied with dementia (Gomperts et al., 2013). In this study, we found plasma amyloid β was significantly higher in PDS compared with controls, consistent with those reported in previous studies (Seino et al., 2019). Moreover, our observations that plasma Aβ42/40 level positively correlated with global cognitive status in patients with APD further confirmed the close connection between amyloid β and cognitive dysfunction. However, another finding revealed that plasma Aβ42 levels were positively correlated to UPDRS part III in patients with MSA, despite no enough evidence for the correlation at present.

The major component of neurofibrillary tangles is the natively unfolded phosphoprotein tau. In human neurodegenerative diseases with filamentous deposits, such as AD, tau protein becomes hyperphosphorylated, as an initiating event preceding filament assembly (Lee et al., 2001). The imbalance of the tau isoforms with three (3R-tau) or four (4R-tau) microtubule-binding repeat domains contributes to the pathogenesis of neurodegeneration. Particularly, PSP is a 4R tau neuropathological entity. Several studies have reported that elevated total tau levels in CSF may help to distinguish PDSs (Süssmuth et al., 2010; Aerts et al., 2011), whereas no such comparable findings were observed in blood (Lin et al., 2018; Li et al., 2021). Furthermore, total tau may be an indicator of cognition. Total tau protein levels have been applied to discriminate dementia with Lewy bodies and PD dementia (PDD) from AD (Howard et al., 2021). However, in our study, measurement of plasma total tau alone was not able to achieve good diagnostic value. Possible explanations may be due to the limited number of patients enrolled and deficient measurement of phosphorylated tau protein.

Our results highlight that plasma NfL is an extraordinarily prospective biomarker as a single measure for differentiating PDSs, whereas other plasma biomarker measurements could not reach an ideal diagnostic value. However, a panel of plasma biomarkers was proven to make great improvement in diagnostic accuracy. A combination of α-syn, Aβ42, Aβ40, Aβ42/40, and NfL could achieve a best diagnostic value in differentiating PDS from HC and PD from HC. As we speculated, by adding NfL to measurements of other biomarker models, there was an obvious improvement in differentiating APD patients from PD and HC. When adding NfL to measurements of α-syn, Aβ42, Aβ40, or Aβ42/40, the best discriminating panel was formed in differentiating APD and HC with a sensitivity of 100% and specificity of 100%, and combination of NfL, Aβ42, and total tau was proved to be the most reliable panel for differential diagnosis between PD and APD, with equally high diagnostic accuracy. For differentiating the subtypes of APD from one another, our results revealed that measurement of NfL, total tau, Aβ42, Aβ40, and Aβ42/40 was the best discriminating panel. Our findings of improved discriminatory potential using a biomarker panel confirm that the combination of NfL with other plasma biomarkers could differentiate PDSs more accurately than a single blood biomarker.

The major strength of our present study was that we evaluated the diagnostic value of several specific plasma biomarkers (NfL, total tau, Aβ42, Aβ40, Aβ42/40 and α-syn) for discriminating PDSs. Combining multiple blood-based biomarkers considerably increases the diagnostic sensitivity and specificity. Simultaneously, we conducted the correlation analysis of plasma biomarkers with motor symptom and cognition. However, there are some limitations to our present study. First, final diagnoses of our participants were established based on clinical evaluations rather than neuropathological confirmation, which increased the possibility of misclassification. Second, the relatively restricted number of patients and lack of follow-up and tracking the changes of biomarkers over time may limit the clinical application to PDS patients. Third, there were significant differences in clinical characteristics among different groups, for which we interpret that the diagnostic groups we enrolled are heterogeneous; namely, the disease severity is diverse among different PDSs, embodied in motor symptoms (H&Y stage, MDS-UPDRS III) and non-motor features (cognitive impairment, sleep disorders, and mood disturbances). For this reason, bias may arise. Finally, a combination of other candidate biomarkers, including CSF, serum, saliva, and skin, would be an enhancement in diagnosis accuracy.

## Conclusion

Our findings support the use of a panel of biomarkers to differentiate PDSs from HC, APD from PD, and the subtypes of APD from one another. Plasma NfL is an extraordinarily prospective biomarker contributing to a biomarker panel in discriminating APD patients from PD and HC and discriminating different subtypes of APD. This outcome may have essential clinical benefit in terms of improving diagnostic accuracy of PDSs. Further large-scale, longitudinal follow-up studies are needed to evaluate the differential value of these integrated plasma biomarkers and validate whether plasma biomarkers levels could be applied to predict progression of motor and non-motor symptoms in patients with PDSs.

## Data Availability Statement

The original contributions presented in the study are included in the article/supplementary material, further inquiries can be directed to the corresponding author.

## Ethics Statement

The studies involving human participants were reviewed and approved by the Ethics Committee of the General Hospital of Tianjin Medical University. The patients/participants provided their written informed consent to participate in this study.

## Author Contributions

QL: conceptualization, methodology, software, formal analysis, writing – original draft, and writing – review and editing. ZheL: investigation, resources, methodology, data curation, software, and writing-original draft. XH, XS, FW, LB, ZhuL, RZ, and YW: investigation, validation, and writing – review and editing. XZ: supervision, project administration, and funding acquisition. All authors read and approved the final version of the manuscript.

## Conflict of Interest

The authors declare that the research was conducted in the absence of any commercial or financial relationships that could be construed as a potential conflict of interest.

## Publisher’s Note

All claims expressed in this article are solely those of the authors and do not necessarily represent those of their affiliated organizations, or those of the publisher, the editors and the reviewers. Any product that may be evaluated in this article, or claim that may be made by its manufacturer, is not guaranteed or endorsed by the publisher.
